# Spinal Cord Injury and Ageing: The Role of Chronic Neuroinflammation

**DOI:** 10.14336/AD.2025.10630

**Published:** 2026-07-01

**Authors:** Tianwei Wang, Zhaoyang Zhang, Jian Liu, Liping Zhang, Qingbin Ni, Baoliang Sun, Jingyi Sun

**Affiliations:** ^1^Department of Neurology, The Second Afﬁliated Hospital, Shandong First Medical University & Shandong Academy of Medical Sciences, Taian, Shandong, China.; ^2^Institute of Brain Science and Brain-inspired Research, Shandong First Medical University & Shandong Academy of Medical Sciences, Jinan, Shandong, China.; ^3^Department of Obstetrics and Gynaecology, Qilu Hospital of Shandong University; Shandong Key Laboratory of Reproductive Health and Birth Defects Prevention and Control, Jinan, Shandong, China.; ^4^Department of Neurology, Shandong Second Provincial General Hospital, Jinan, Shandong, China.; ^5^The Affiliated Taian City Central Hospital of Qingdao University, Taian, Shandong, China.; ^6^Department of Spinal Surgery, Shandong Provincial Hospital Affiliated to Shandong First Medical University, Jinan, Shandong, China

**Keywords:** aging, spinal cord injury, chronic neuroinflammation, immunosenescence, inflammasome

## Abstract

As the global population ages, there is an increasing prevalence of spinal cord injury (SCI) among elderly individuals, accompanied by significant challenges in treatment and recovery. Age-related conditions, such as osteoporosis, muscle atrophy, and impaired balance, predispose older adults to falls and traumatic injuries, leading to worse neurological outcomes compared to younger patients. SCI pathophysiology consists of two phases: the initial mechanical injury and the secondary injury that involves a cascade of pathological events, including ischemia, apoptosis, and neuronal cell death. Chronic neuroinflammation has emerged as a central factor in driving long-term damage after SCI, particularly in older people, where immune senescence and a decreased ability to resolve inflammation contribute to persistent, unresolved inflammation. This prolonged inflammatory state further impedes neural regeneration and functional recovery. Aged animal models have revealed that chronic neuroinflammation is exacerbated by sustained activation of microglia and astrocytes, the infiltration of peripheral immune cells, and the secretion of pro-inflammatory cytokines, creating a pro-inflammatory microenvironment that hinders repair. Furthermore, ageing-related factors such as immunosenescence, autophagy dysfunction, and mitochondrial abnormalities exacerbate inflammation, establishing a vicious injury cycle. Despite promising studies targeting inflammation in young SCI models, there is a critical need for age-specific therapeutic approaches for elderly SCI patients. This review explores the mechanisms of chronic inflammation in aged SCI, examines key cellular mediators, and discusses potential therapeutic strategies, including pharmacological treatments, gene therapy, exosome-based interventions, and rehabilitation. Focusing on age-related differences in inflammation and healing, this work aims to provide a foundation for precision medicine tailored to the ageing population with SCI, ultimately improving clinical outcomes and quality of life for elderly patients.

## 1. Introduction

With the accelerating global trend of population aging, health issues among the elderly have become a central focus of medical research. By 2050, individuals aged 65 and older are projected to account for 20% of the global population, with the number of those aged 80 and above expected to triple [1, 2]. The growing elderly population has led to a marked increase in the incidence of SCI [3]. Due to age-related conditions such as osteoporosis, muscle atrophy, and impaired balance, older adults are more susceptible to falls and trauma [4]. Consequently, they face a higher risk of complications and exhibit poorer neurological recovery compared to younger patients.

Traditionally, the pathophysiological process of SCI consists of two main phases: the initial mechanical insult and the subsequent secondary injury. The secondary phase encompasses a cascade of pathological events, including ischemia, apoptosis, and neuronal cell death. However, a growing body of evidence suggests that chronic neuroinflammation may serve as a central driving force in the long-term pathological progression of SCI [5]. This phenomenon is particularly pronounced in elderly patients. Elderly individuals often exhibit a persistent, unresolved state of chronic inflammation due to immune senescence and impaired inflammation resolution mechanisms [6]. This sustained inflammation causes direct damage to neural tissue. It interacts with age-related pathological changes—such as mitochondrial dysfunction, oxidative stress, and impaired autophagy—forming a vicious cycle that further suppresses neural regeneration [7-9].

However, current research predominantly relies on young animal models, which not only overlooks the unique role of aging in SCI but also leads to a significant gap in understanding the mechanisms of chronic inflammation in the context of aged SCI [10]. Although young animal models play a crucial role in preliminary studies, they often fail to accurately represent the unique physiological changes experienced by elderly SCI patients [8]. The decline in immune system function, the coexistence of chronic diseases, and the different response mechanisms to injury in elderly individuals mean that research overly reliant on young animal models lacks broad applicability to treatment strategies for the elderly population. This issue affects the accuracy of pathological research and leads to biases in developing clinical treatment strategies. Therefore, it is crucial to emphasise the study of inflammation mechanisms in aged animal models and the development of standardised aged SCI models.

Due to the profound understanding of the limitations of young animal models, current research has begun to reveal that chronic neuroinflammation in aged SCI is not merely a passive response to injury, but may actively drive disease progression. Elderly patients with SCI and aged animal models exhibit prolonged activation of microglia and astrocytes, accompanied by the sustained release of pro-inflammatory cytokines such as IL-1β, TNF-α, and IL-6 [6]. This is further exacerbated by the infiltration of peripheral immune cells, which amplifies local inflammation. Although animal studies suggest that eliminating activated glial cells may enhance neural regeneration following SCI, this therapeutic approach has yet to be successfully translated into clinical practice [11]. Moreover, existing anti-inflammatory drugs have not been approved explicitly for targeting chronic inflammation in elderly patients with SCI, highlighting a critical unmet need for age-specific therapeutic interventions [12].

Therefore, elucidating the specific mechanisms of chronic inflammation in the context of aging-related SCI and exploring effective intervention strategies is critical. This review systematically examines the role of chronic neuroinflammation following SCI in aged individuals, focusing on key cellular players, molecular pathways, and their impact on disease progression. We also highlight current research gaps and challenges and discuss potential therapeutic approaches—including pharmacological treatments, cell and gene therapies, and rehabilitative interventions—to provide a theoretical foundation and translational basis for precision medicine in elderly patients with SCI.

## 2. Chronic Neuroinflammation in the Aged Spinal Cord

### 2.1 Persistence of Inflammation

The inflammatory response following SCI is a highly dynamic process, with its progression from acute onset, peak, to resolution (or chronicization) playing a decisive role in the evolution of neural damage and the potential for repair (Table 1). Table 1 summarizes the inflammatory response at different stages following SCI. The entire inflammatory process is divided into three main phases: the initiation phase (0-24 hours), the peak phase (1-7 days), and the resolution/chronic phase (2-4 weeks and beyond). Each phase lists the key immune cells involved, such as microglia, astrocytes, and neutrophils, as well as major pro-inflammatory cytokines expressed during these periods, including IL-1β, TNF-α, and IL-6. The table clearly illustrates the dynamic changes in inflammation after SCI and highlights the roles of different immune cells and cytokines at each stage. Notably, this process is more likely to evolve into a persistent chronic inflammatory state in the context of aging.

### 2.1.1 Onset phase (0-24 hours post-injury)

Injury immediately triggers a strong acute inflammatory response. Damage-associated molecular patterns (DAMPs) released from neuronal damage rapidly activate resident immune cells, particularly microglia and astrocytes [13]. Activated glial cells secrete many pro-inflammatory cytokines (such as IL-1β, TNF-α, and IL-6), significantly amplifying the inflammatory cascade by activating signalling pathways like NF-κB [14]. As the earliest responding peripheral immune cells, neutrophils begin to infiltrate the core injury area within 4-6 hours post-injury, becoming the primary pro-inflammatory effector cells at this stage [15]. They further secrete TNF-α and IL-1β and recruit subsequent monocytes and macrophages [16].

**Table 1:** Overview of Inflammatory Stages Following SCI and Key Immune Cells and Cytokines.

Stage	Time window	Key immune cells	Key pro-inflammatory cytokines	References
**Onset phase**	0-24 hours	-Microglia (rapid activation) -Astrocytes (activation) -Neutrophils (infiltrate within 4-6 hours)	-IL-1β, TNF-α, IL-6	[13-16]
**Peak phase**	1-7 days	-Neutrophils (early peak) -Monocytes/macrophages (massive infiltration after a few days) -Astrocytes (proliferate within 1-2 days, glial scar formation by 7-9 days)	-IL-1β (peaks later) -TNF-α (peaks around 36 hours) -IL-6(delayed high expression)	[13, 17-20]
**Resolution/Chronic phase**	≥2-4 weeks	-Microglia (persistent activation) -Astrocytes (persistent activation) -Astrocytes (persistent activation)	-IL-1β, IL-6 (persistently elevated) -TNF-α (detectable for extended periods)	[13, 21-24]

### 2.1.2 Peak phase (1-7 days post-injury)

The inflammatory response reaches its peak during this phase. Neutrophil numbers peak in the early stages post-injury, followed by a significant infiltration of monocytes and macrophages several days later, which form the majority of the inflammatory cell infiltrate, driving the inflammation to its peak [17]. The expression levels of key pro-inflammatory cytokines, including IL-1β, TNF-α, and IL-6, also peak during this period [18]. It is essential to emphasise the differences in the expression dynamics of various pro-inflammatory cytokines: TNF-α typically peaks rapidly around 36 hours post-injury, while the high expression of IL-1β and IL-6 occurs with a relative delay [13]. Meanwhile, astrocytes proliferate significantly within 1-2 days post-injury, forming a dense glial scar structure by approximately 7-9 days [19]. Although the glial scar physically limits the spread of the injury area, the excessive secretion of axon growth inhibitors, such as chondroitin sulfate proteoglycans (CSPGs), forms a chemical barrier that hinders neural regeneration [20].

### 2.1.3 Resolution/Chronic phase (2-4 weeks post-injury and beyond)

In ideal conditions or among young individuals, the inflammatory response should gradually resolve during this phase: the activation of immune cells diminishes, levels of pro-inflammatory cytokines decrease, and repair processes begin to predominate [13, 21]. However, in reality, especially in aged individuals, inflammation often fails to resolve effectively and instead persists, entering a chronic phase. Although the blood-brain barrier gradually repairs during this phase, the persistent activation of glial cells (microglia and astrocytes) becomes a key factor in sustaining chronic inflammation [22]. Experimental evidence demonstrates the long-term nature of inflammation; for example, in the T12 contusive SCI model, Iba1-positive activated microglia and macrophages persist from 1 to 12 weeks post-injury [23]. Their activation signals can still be detected in the dorsal and ventral white matter regions 5 mm above the injury centre [23]. Similarly, in the compression SCI model, the pro-inflammatory cytokines IL-1β and IL-6 remain significantly elevated at 28 days post-injury [24]. This long-term, sustained activation of immune cells and release of inflammatory cytokines profoundly negatively impacts the neural repair process. Notably, in aged SCI models, the resolution of inflammation is significantly delayed and more intense, characterized by prolonged immune cell activation and higher levels of pro-inflammatory cytokines.

### 2.1.4 Mechanisms of chronic inflammation maintenance

The persistence of chronic inflammation is closely linked to the dysfunction of glial cells. In a chronically inflamed environment, microglia tend to shift from a reparative M2-like phenotype toward a pro-inflammatory M1-like state [25]. This transition results in the sustained release of neurotoxic mediators, such as reactive oxygen species (ROS), nitric oxide, and IL-1, thereby establishing a self-perpetuating inflammatory cycle that further impedes tissue repair [26]. Meanwhile, activated astrocytes promote glial scar stabilisation by secreting chemokines and extracellular matrix components (such as collagen and proteoglycans), further exacerbating inflammation and hindering regeneration [27]. This results in the so-called "gliopathy," a dysfunction that astrocytes exhibit in a chronic inflammatory state. The characteristic features of this pathology include persistent activation of astrocytes, excessive secretion of pro-inflammatory cytokines, and abnormal proliferation and migration [28]. These changes intensify the local inflammatory response and interfere with neural repair.

In summary, inflammation following SCI is not a transient event, but a chronic process driven by sustained glial activation, self-perpetuating pro-inflammatory cytokine release, and glial scar barrier formation. Without appropriate intervention, this pathological microenvironment exacerbates secondary injury and significantly impairs functional recovery. More critically, inflammation's intrinsic "resolution mechanisms" are profoundly compromised under aging conditions, leading to intensified and persistently unresolved inflammatory responses (Section 2.2).

### 2.2 Aging Exacerbates the Inflammatory Response

**Figure 1.**
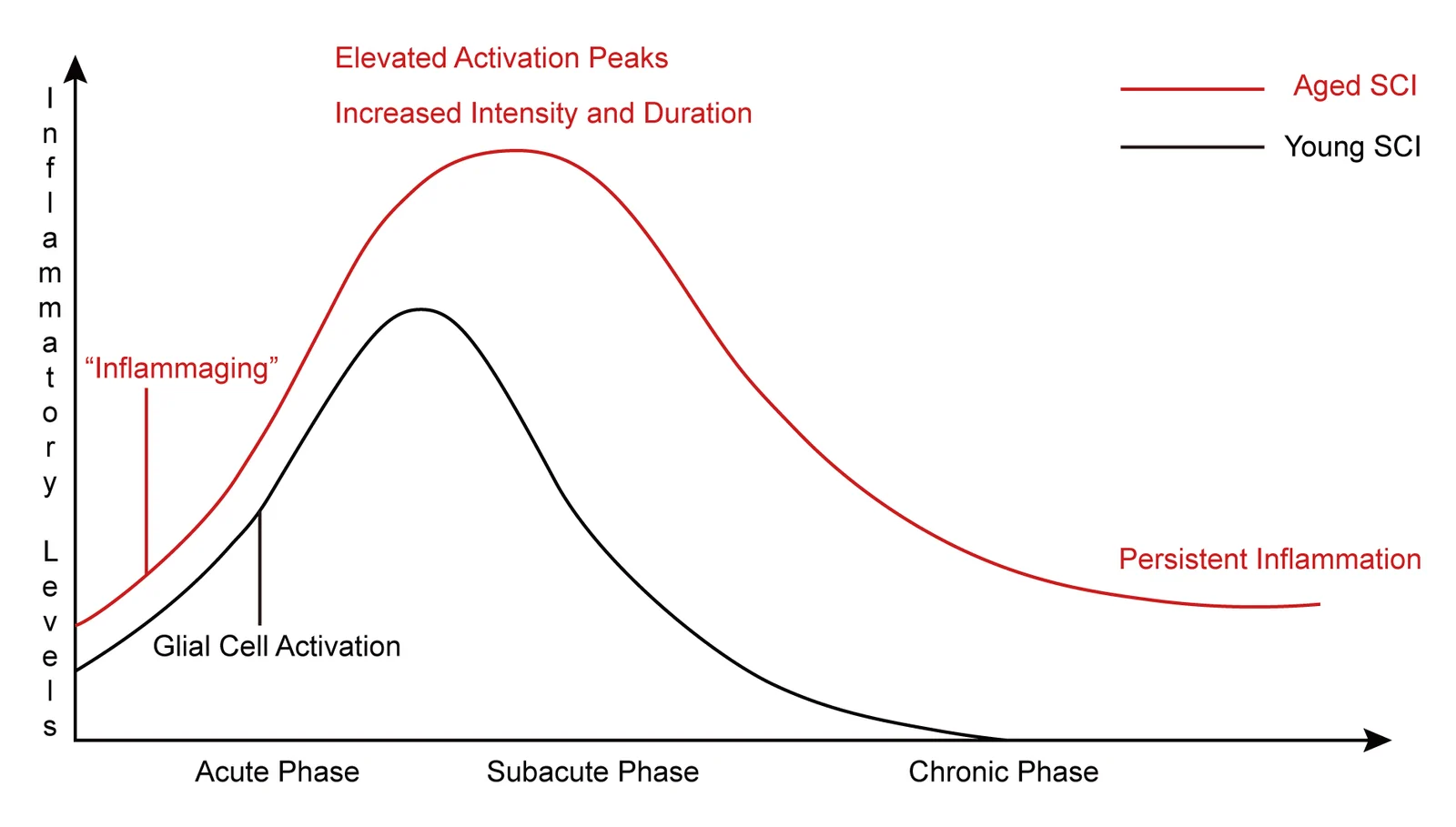
**Comparison of inflammatory response trajectories following SCI in aged versus young individuals**. The figure illustrates that the aged spinal cord exhibits a higher baseline inflammatory state, termed "inflammaging," resulting in elevated activation peaks, increased intensity, and prolonged duration of inflammation compared to young spinal cords. Unlike the younger group, aged individuals exhibit persistent, unresolved chronic inflammation, highlighting age-related vulnerability to sustained neuroinflammatory damage after SCI.

Aging systemically remodels the neuroimmune microenvironment, rendering elderly individuals more susceptible to high-intensity and persistent chronic inflammation following SCI (Fig. 1) [29]. The underlying mechanisms include:

### 2.2.1 Lowered Baseline Inflammatory Threshold (“Inflammaging”)

Even in the absence of overt injury, the neuroimmune microenvironment of the aged spinal cord exhibits marked alterations. Microglia adopt a characteristic "primed" phenotype, marked by hypertrophic cell bodies and reduced process complexity, and persistently overexpress pro-inflammatory cytokines such as TNF-α and IL-1β [30, 31]. Concomitantly, baseline infiltration of T lymphocytes and monocytes is increased in both the perivascular spaces and the spinal cord parenchyma [32, 33]. This chronic, low-grade inflammatory state—often termed "inflammaging"—reflects a fundamental disruption of neuroimmune homeostasis, resulting in a significantly lowered threshold for activation of post-injury immune responses. Experimental studies have shown that injurious stimuli of equivalent intensity evoke approximately a two-fold increase in pro-inflammatory cytokine release in the aged spinal cord compared to young controls, accompanied by a more rapid onset of the inflammatory response [34]. This "sensitised" state renders the aged spinal cord abnormally prone to even minor insults, readily breaching physiological regulatory thresholds and precipitating uncontrolled inflammatory cascades.

### 2.2.2 Markedly Increased Intensity and Prolonged Duration of the Inflammatory Response

Compared with young animals, aged animals exhibit a more intense and prolonged inflammatory response following SCI. Histological and molecular analyses have demonstrated that, following SCI, aged mice exhibit more severe tissue damage and functional deficits, accompanied by prolonged upregulation of inflammation-related mediators, indicative of impaired inflammation resolution [8]. RNA-seq analysis further revealed that, in the aged spinal cord, the "microglial activation" and "autophagy dysregulation" pathways remain persistently upregulated, and there is a pronounced accumulation of myeloid immune cells within the lesion core [35]. Single-cell transcriptomic analysis has confirmed that the aged spinal cord primarily harbours a highly pro-inflammatory microglial subset, contrasting with the predominantly resting or mildly pro-inflammatory cells in young animals [36]. This subset exhibits higher expression levels of Nos2 and Il1b, along with enhanced immune signalling capabilities. These highly pro-inflammatory microglial subsets are classified as the "B type" phenotype [36]. In aged mice, B-type microglia exhibit enhanced pro-inflammatory characteristics, including elevated Nos2 and Il1b expression levels and stronger immune signalling capabilities. Aging amplifies the overall magnitude of microglial responses. It drives their transition toward a refractory proinflammatory phenotype, thereby prolonging and exacerbating local chronic inflammation and imposing a sustained impediment to neural repair.

### 2.2.3 Immunosenescence and Cellular Senescence Amplify Inflammation

Aging further elevates chronic inflammation levels through the synergistic interaction between "immunosenescence" and "cellular senescence." This synergistic effect manifests as a progressive decline in immune system function, which, in concert with the accumulation of senescent cells within tissues, mutually amplifies the inflammatory signaling cascade. Firstly, immunosenescence skews macrophage/microglial and T-cell phenotypes toward a pro-inflammatory state, with a marked decline in the synthesis and secretion of anti-inflammatory mediators, such as IL-10, thereby impairing the clearance and resolution of inflammation [37]. Meanwhile, a substantial accumulation of senescent cells within the spinal cord persistently releases senescence-associated secretory phenotype (SASP) factors, such as CCL11, IL-6, IL-8, and various matrix metalloproteinases (MMPs)[38]. These components create a high-concentration "cloud" of chemokines and cytokines that chronically recruit and activate surrounding myeloid and lymphoid immune cells [39].

### 2.2.4 Autophagy and Mitochondrial Dysfunction

In the context of SCI, autophagy dysfunction and mitochondrial abnormalities—both hallmarks of the Aging process—play critical roles in driving and sustaining chronic neuroinflammation. Studies have shown that mitochondrial damage is exacerbated following SCI in aged individuals, leading to excessive production of ROS and the release of mitochondria-derived DAMPs [40]. These signals persistently activate pro-inflammatory pathways, such as NF-κB, amplifying the local inflammatory response. Meanwhile, impaired autophagy hinders the timely clearance of damaged organelles and inflammatory mediators, allowing inflammatory signalling and oxidative stress to exacerbate each other. This interplay establishes a vicious cycle of "inflammation-oxidative stress," which further exacerbates neuronal injury and hinders functional recovery.

In summary, Aging markedly amplifies chronic inflammation following SCI, evidenced by a lowered baseline inflammatory threshold, heightened inflammatory intensity, prolonged duration, and more persistent, less reversible immune cell phenotypes. Multiple aging-related factors, including immuno-senescence, cellular senescence, impaired autophagy, and mitochondrial dysfunction, act synergistically to create a pro-inflammatory environment, in which elderly individuals are more prone to enter a state of unresolved, chronic inflammation after SCI. Therefore, elucidating the specific mechanisms by which aging modulates post-injury inflammation is crucial for developing targeted therapeutic strategies tailored to the elderly population and improving clinical outcomes.

## 3. Cellular Mediators of Inflammation

**Figure 2.**
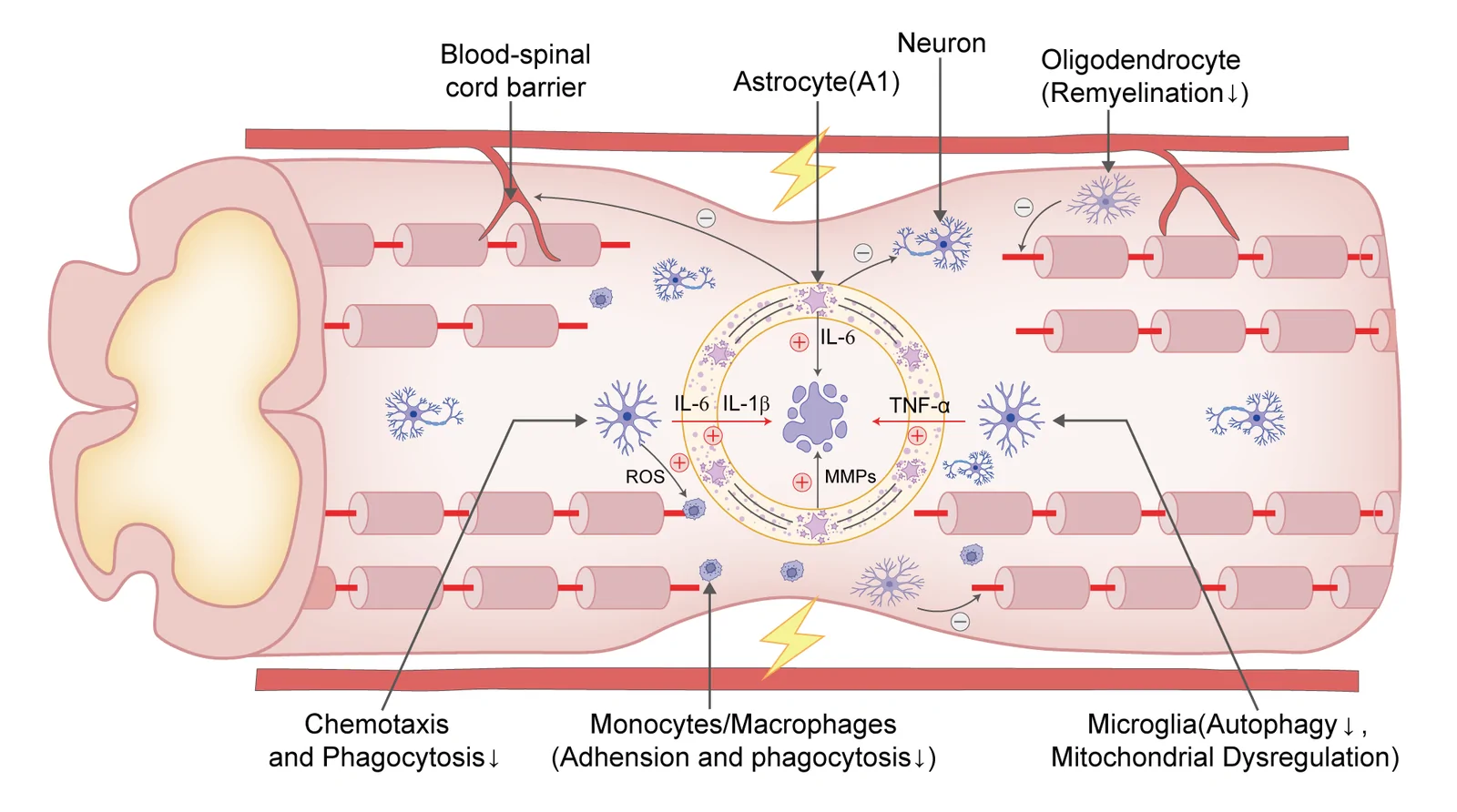
**Cellular Mechanisms of Inflammation and Repair Following SCI: Impacts of Aging on Immune and Glial Cell Functions**. This figure illustrates the roles of microglia, astrocytes, monocytes/macrophages, and oligodendrocytes in SCI during aging and their contributions to exacerbated inflammatory responses. The schematic demonstrates that these senescent cells, upon injury, excessively secrete pro-inflammatory cytokines such as IL-1β, IL-6, TNF-α, and ROS, thereby promoting sustained and expanded inflammation. With advancing age, the functional capacities of these cells become compromised, including impaired chemotaxis and phagocytosis, leading to inefficient clearance of cellular debris in the lesion area. This dysfunction further amplifies neuroinflammation and hinders effective neural repair.

In the inflammatory response following SCI, the activation of immune cells and reactive changes play a critical role. Microglia, astrocytes, oligodendrocytes, and peripheral immune cells drive the expansion and repair of neural injury through the secretion of pro-inflammatory cytokines and participation in cellular responses. Especially in aging individuals, the dysfunction of these cells and excessive inflammatory reactions significantly hinder injury repair and functional recovery, further exacerbating chronic neuroinflammation (Fig. 2).

### 3.1 Microglia

Microglia are the resident immune cells of the central nervous system (CNS) and play a crucial role in secondary injury following SCI [41]. Under normal conditions, microglia are rapidly activated after injury, releasing pro-inflammatory cytokines (such as IL-1β, TNF-α, and IL-6) that trigger a local inflammatory response [14, 42]. However, in aged individuals, the state of microglia undergoes significant changes, manifesting as a "primed" or pre-activated state. Cells in this state exhibit more vigorous baseline inflammatory activity and respond more intensely to stimuli [43]. The secretion of pro-inflammatory cytokines (such as IL-1β and TNF-α) by aged microglia is significantly increased [44]. However, their chemotactic and phagocytic functions are impaired [44]. This results in a persistent inflammatory response that hinders the repair process of the injury. Recent studies have revealed that impaired autophagy and mitochondrial dysfunction in aged microglia are key factors driving their prolonged activation and the maintenance of chronic inflammation [45]. Impaired autophagy prevents cells from effectively clearing damage and toxic substances, leading to the sustained activation of the NLRP3 inflammasome [46]. For example, Beclin1-deficient mice exhibit a stronger microglial inflammatory response after SCI, producing more pro-inflammatory cytokines and exacerbating neural damage [46]. On the other hand, with the decline in mitochondrial function, the accumulation of ROS significantly enhances the activation of the NLRP3 signalling pathway, further promoting the maturation and release of IL-1β, thereby intensifying the inflammatory response [47]. The autophagy-mitochondrial dysfunction and NLRP3 inflammasome vicious cycle in aged microglia constitutes the core mechanism of sustained inflammation in chronic SCI, significantly hindering neural regeneration and functional recovery.

In summary, aged microglia maintain chronic inflammation through persistent activation and dysfunction, becoming a significant obstacle to neural repair following SCI. Interventions targeting this mechanism may provide new therapeutic avenues for elderly SCI patients.

### 3.2 Astrocytes and Glial Scar Formation

Astrocytes constitute the principal cellular component of the glial scar that forms following SCI. After the injury, astrocytes are stimulated by cytokines and damage-associated signals to proliferate and migrate to the lesion site, where, together with oligodendrocyte precursor cells and microglia, they encircle the lesion core and contribute to glial/fibrotic scar formation [48]. In these peri-lesional zones, astrocytes actively remodel the extracellular matrix by upregulating components that inhibit axonal regeneration, such as CSPGs, and by secreting abundant matrix molecules, including collagen and fibronectin, thereby establishing physical and chemical barriers that constrain the spread of inflammation but simultaneously impede neural regeneration [22, 49].

Aging markedly augments astrocyte reactivity and enhances their molecular output. In aged individuals, astrocytes shift toward the A1 neurotoxic phenotype, exhibiting upregulated expression of activation markers, such as GFAP and vimentin [50]. At the same time, senescent cells exhibit a typical SASP, secreting numerous inflammatory mediators [51]. These mediators activate inflammatory signaling pathways in the neuronal microenvironment and profoundly disrupt the perivascular milieu. Studies have shown that in A1-type astrocytes, the NF-κB signaling pathway is activated, driving the robust release of inflammatory mediators such as IL-6 and MMP-3, which exert cytotoxic effects on neurons, blood-brain barrier endothelial cells, and pericytes [52]. In aged brain-injury models, elevated MMP-9 activity correlates with increased blood-brain barrier permeability, indicating that the BBB is more vulnerable to injury in the elderly [53].

In summary, astrocytes play a pivotal role after SCI through glial scar formation and the regulation of inflammation, whereas aging markedly amplifies their pathological activation, resulting in excessive release of inflammatory mediators, exacerbation of the inflammatory milieu, disruption of blood-brain barrier integrity, and ultimately profound inhibition of neural repair and functional recovery. This mechanism may constitute a key contributor to the poor prognosis observed in elderly patients with SCI.

### 3.3 Other Cell Types: Oligodendrocytes and Peripheral Immune Cells

Oligodendrocytes are the myelinating cells of the CNS and, following SCI, contribute to axonal remyelination by differentiating into newly myelinating oligodendrocytes. In adult individuals, SCI induces the proliferation of NG2^+^ oligodendrocyte precursor cells, which migrate to the lesion site and differentiate into myelinating oligodendrocytes to repair the disrupted myelin sheaths [54]. However, aging severely impairs this remyelination capacity. Recent non-human primate studies have demonstrated a marked reduction in the number of newly generated myelinating oligodendrocytes in aged monkey brains, a phenomenon not due to inadequate precursor cell proliferation but rather to impaired differentiation capacity, as reflected by downregulated lineage-specific transcription factors and diminished expression of myelin proteins [55]. These alterations result in delayed and incomplete myelin repair in the aged spinal cord, leading to persistent deficits in neural conduction. In the context of SCI, similar age-related changes may impair oligodendrocyte-mediated compensation for demyelinating injury, thereby exacerbating chronic pathology.

Peripheral immune cells, including monocytes/macrophages and lymphocytes, also massively infiltrate the lesion site following SCI [21]. Meanwhile, helper T cells and cytotoxic T cells also accumulate at the lesion site, mediating adaptive immune responses [56]. However, aging markedly alters the behaviour of these immune cells. Aged peripheral monocytes and macrophages exhibit impairments in chemotaxis, adhesion, and phagocytosis; for example, reduced L-selectin expression on monocytes in elderly individuals limits their migratory capacity toward inflammatory foci [44]. Conversely, upon stimulation by pathogens or injury, these cells secrete markedly elevated levels of pro-inflammatory mediators such as TNF-α. In SCI animal models, macrophages from aged mice exhibit enhanced M1 polarisation—producing elevated levels of IL-1β, TNF-α, and iNOS—while regulatory IL-4 signalling is attenuated, resulting in persistent inflammation that fails to resolve spontaneously [57, 58]. Furthermore, aging markedly alters the composition and functional characteristics of T-cell subsets, as manifested by an increased proportion of memory-phenotype helper T cells and a diminished immunosuppressive capacity of regulatory T cells (Tregs), thereby precipitating a dysregulated peripheral immune response to CNS injury signals [59, 60]. In summary, in aged individuals, the impaired chemotaxis, polarisation, and phagocytic functions of immune cells result in uncontrolled post-SCI inflammatory responses, exacerbating chronic neuroinflammation and hindering injury repair.

In summary, aging not only markedly impairs oligodendrocyte differentiation and remyelination capacity, resulting in persistent deficits in neural conduction, but also disrupts the chemotactic, polarisation, and phagocytic functions of peripheral immune cells, manifested by sustained pro-inflammatory mediator release and weakened anti-inflammatory signaling, thereby precipitating uncontrolled chronic neuroinflammation. These cellular alterations collectively hinder tissue repair and functional recovery after SCI and represent a key contributor to the poor prognosis and delayed recovery observed in elderly SCI patients.

## 4. Key Molecular Pathways Linking SCI, Aging, and Chronic Inflammation

**Figure 3.**
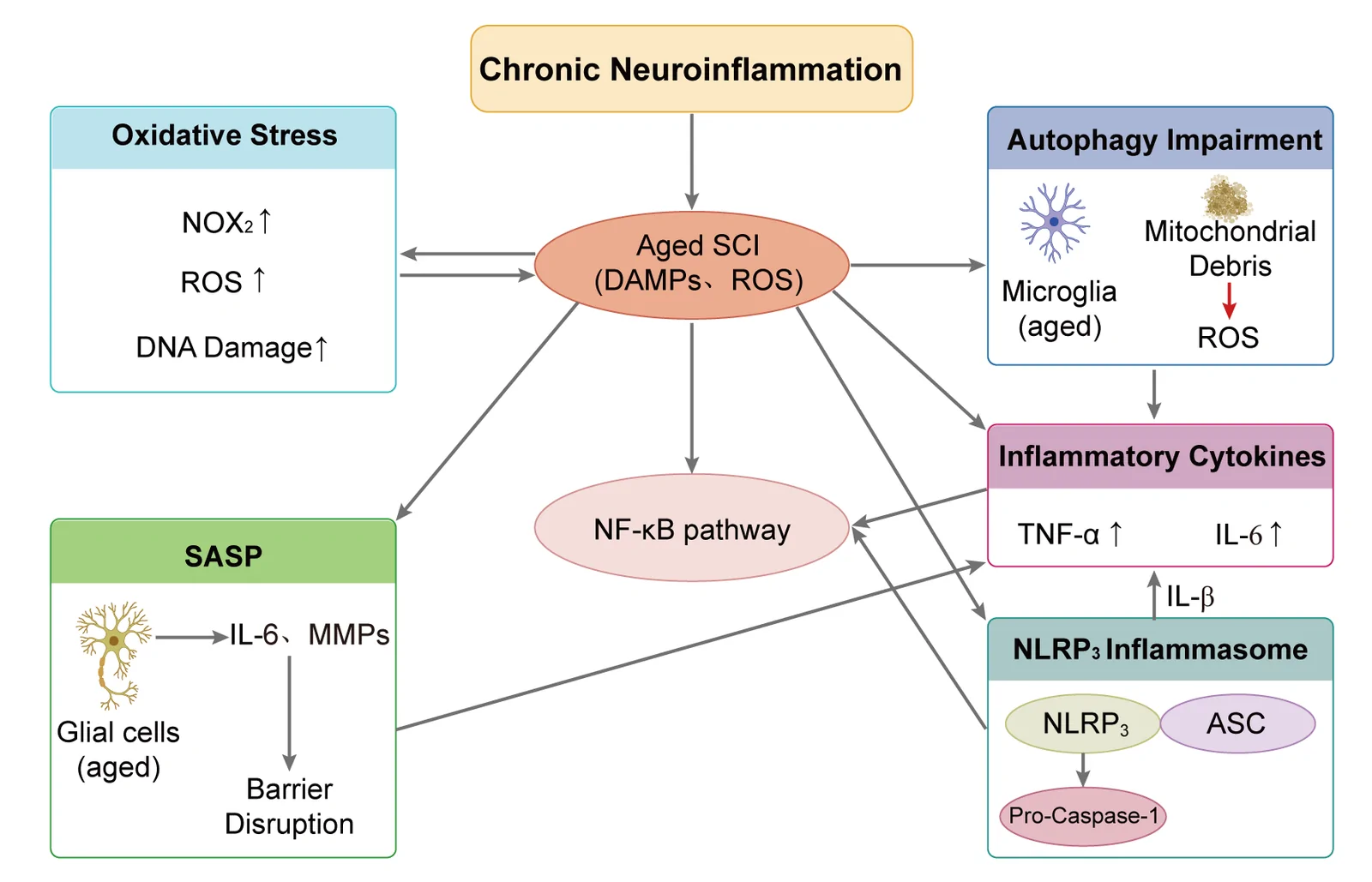
**Molecular Pathways Driving Chronic Neuroinflammation in Aged SCI**. This figure outlines the key molecular mechanisms underlying chronic neuroinflammation following SCI in the aged population. It illustrates how oxidative stress, the SASP, impaired autophagy, the release of pro-inflammatory cytokines, and activation of the NLRP3 inflammasome interact primarily through the NF-κB signaling pathway to drive sustained inflammatory responses. In the aged spinal cord post-injury, the accumulation of DAMPs and ROS initiates these interconnected signaling cascades, leading to persistent damage and inflammatory activation of neurons and glial cells. These pathological processes collectively contribute to chronic tissue degeneration and impaired regeneration in the aging spinal cord.

In the aging setting, a network of molecular pathways activated following SCI converges to drive chronic neuroinflammation. These principally include NLRP3 inflammasome activation, pro-inflammatory cytokine release via NF-κB signaling, oxidative stress, autophagy dysregulation with impaired proteostasis, and the SASP. Below, we systematically outline the defining characteristics of each pathway and their interrelationships. As shown in Figure 3, the interactions of these pathways play a crucial role in chronic neuroinflammation following SCI in aging individuals.

### 4.1 NLRP3 Inflammasome

In the aged context, a cascade of molecular pathways triggered by SCI collectively drives chronic neuroinflammation, activating the NLRP3 inflammasome as a critical initiating event. The NLRP3 inflammasome is a multiprotein complex of the innate immune system, comprising the sensor molecule NLRP3, the adaptor protein ASC, and the zymogen pro-caspase-1 [61]. DAMPs and ROS function as the "signal 1" priming event for NLRP3 transcription in microglia and can further potentiate the inflammatory response by directly activating the NF-κB pathway. Upon recognition of DAMPs, the PYD domain of NLRP3 associates with the PYD of ASC, after which ASC's CARD domain recruits pro-caspase-1 to assemble the full NLRP3-ASC-pro-caspase-1 complex. Activated caspase-1 cleaves pro-IL-1β and pro-IL-18 into their mature forms, initiating an inflammatory cascade and ultimately inducing pyroptotic cell death [62-64]. Activated caspase-1 also potentiates NF-κB signalling, establishing a self-amplifying feed-forward loop that, through sustained release of cytokines and cellular debris, further promotes NLRP3 inflammasome activation. In summary, in the context of aging, SCI robustly engages the NLRP3 inflammasome by sensing DAMPs and ROS, driving the maturation and release of pro-inflammatory cytokines and triggering pyroptosis. Moreover, the positive feedback interplay between caspase-1 and NF-κB signaling further amplifies chronic neuroinflammation.

### 4.2 Inflammatory Cytokines

After SCI, pro-inflammatory cytokines such as tumor necrosis factor-alpha (TNF-α) and interleukin-6 (IL-6) are abundantly released at the lesion site and remain persistently elevated. Studies have shown that with increasing age, following SCI, microglia exhibit a pronounced shift toward pro-inflammatory M1 polarisation, with canonical markers such as CD86 and the pro-inflammatory cytokine TNF-α expressed significantly higher levels than in young animals [29]. Furthermore, IL-1β, matured via NLRP3 inflammasome activation, further drives the sustained secretion of IL-6 and TNF-α, resulting in prolonged exposure of peri-lesional tissues to a high-concentration pro-inflammatory milieu [65]. These inflammatory mediators exert pronounced cytotoxic effects on adjacent neurons and glial cells, persistently activating the NF-κB signalling pathway, amplifying pro-inflammatory cytokine release, and establishing a vicious feed-forward loop. Studies have reported that within days after SCI, IL-6 and TNF-α levels rise sharply and persistently into the chronic phase [66]. In aged SCI models, this pro-inflammatory milieu is particularly refractory to resolution, ultimately resulting in exacerbated neuronal apoptosis, demyelination, and fibrotic scar formation [67]. In summary, the sustained overexpression of pro-inflammatory cytokines such as TNF-α and IL-6 establishes the molecular underpinnings of chronic neuroinflammation following SCI.

### 4.3 Oxidative Stress

Following SCI—particularly in aged models—oxidative stress is markedly exacerbated. On the one hand, activated microglia and infiltrating immune cells within the lesion site exhibit elevated expression of NADPH oxidase 2 (NOX2), leading to a significant increase in the production of ROS. Studies have shown that in SCI models of middle-aged rats, NOX2 gene expression in microglia is significantly higher than in young rats, resulting in markedly increased ROS production [29]. This suggests that microglia's age-related "pre-activated" state substantially amplifies the oxidative stress response following injury. On the other hand, excessive accumulation of ROS directly induces oxidative damage to intracellular DNA and proteins. Among these, 8-hydroxy-2'-deoxyguanosine, a well-established marker of DNA damage, is significantly elevated, indicating an exacerbation of oxidative DNA injury in neural cells [29]. ROS-induced cellular damage directly triggers neuronal apoptosis and promotes the release of additional DAMPs, further activating the NLRP3 inflammasome and NF-κB signaling pathways, establishing a vicious cycle between inflammation and oxidative injury. In summary, enhanced NOX2 activity and the resulting overproduction of ROS and oxidative DNA damage in aged SCI models represent key driving forces underlying the persistence of chronic neuroinflammation.

### 4.4 Autophagy and Proteostasis Disruption

Autophagy is a vital cellular process that clears damaged organelles and protein aggregates, thereby preserving proteostasis and cellular homeostasis. Under physiological conditions, autophagic pathways in the injured spinal cord eliminate mitochondrial debris, preventing the excessive activation and accumulation of inflammatory signals [68]. However, aging significantly impairs autophagic function in spinal cord tissue following SCI. A recent study demonstrated that in aged SCI mice, microglia exhibit markedly dysregulated autophagic pathways, characterized by impaired autophagic flux and abnormally reduced expression of autophagy-related proteins [8]. Impaired autophagy leads to the accumulation of damaged mitochondria and protein aggregates, which in turn enhances the production of ROS and inflammatory cytokines, directly contributing to microglial overactivation. Experimental evidence has shown that inhibition of autophagy following SCI significantly enhances the pro-inflammatory response of microglia, whereas promoting autophagic activity—for example, through trehalose treatment—effectively attenuates inflammation and facilitates spinal cord functional recovery [46]. Therefore, the accumulation of mitochondrial debris and protein aggregates resulting from defective autophagy readily amplifies the activation and persistence of local inflammatory signaling, contributing to a vicious cycle of injury. In aged SCI models, autophagic impairment and sustained expression of inflammatory cytokines disrupt proteostasis, exacerbate neuronal damage, and significantly suppress neural regeneration [69]. In summary, aging-induced autophagy dysfunction leads to the accumulation of mitochondrial debris and protein aggregates, promoting the generation of ROS and pro-inflammatory cytokines and excessive microglial activation. This establishes a vicious cycle between inflammation and proteostasis disruption, severely impairing regenerative repair in spinal cord tissue.

### 4.5 SASP Exacerbates Inflammation

With advancing age, astrocytes and microglia within the CNS may enter a state of cellular senescence characterized by acquiring a distinct SASP [70, 71]. Aged glial cells secrete a large amount of pro-inflammatory and pro-fibrotic factors. These SASP factors upregulate pro-inflammatory pathways, such as IL-6/STAT3, and synergize with matrix degradation mediated by MMPs, amplifying the inflammatory response and the scar remodeling process [72]. For example, IL-6, through activating the STAT3 signaling pathway, accelerates the compaction and consolidation of glial scar tissue. In contrast, by degrading extracellular matrix proteins, MMPs compromise the integrity of the blood-spinal cord barrier and further exacerbate fibrosis at the injury site [73]. Animal studies have demonstrated that following SCI, senescent glial cells accumulate prominently at the lesion site, and pharmacological clearance of these cells using senolytic agents effectively reduces scar tissue volume and local inflammatory burden [72]. This functional improvement is accompanied by a marked reduction in the secretion of SASP factors, including IL-6 and MMPs, directly demonstrating the SASP's pivotal role in the SCI recovery process [74]. In summary, in aged SCI models, the SASP displayed by glial cells—through sustained release of pro-inflammatory and pro-fibrotic factors—establishes a self-reinforcing environment of inflammation and scarring that profoundly exacerbates neural damage and inhibits axonal regeneration.

## 5. Immunosenescence and Impaired Inflammation Resolution

With advancing age, immunosenescence not only elevates the risk of infection and malignancy but also profoundly impairs the clearance of inflammation and tissue repair following neural injury. In the SCI context, age-related immune function declines markedly, disrupting post-injury inflammatory regulation and recovery processes. In this section, we will examine in detail how immunosenescence affects inflammatory modulation, barrier integrity, and the mechanisms of inflammation resolution, and we will propose potential intervention strategies to mitigate these deficits.

### 5.1 Immunosenescence

In elderly individuals, immunosenescence is characterized by markedly impaired T-cell and B-cell functions, which constrain post-spinal cord injury (SCI) inflammatory regulation and tissue repair capacity.

### 5.1.1 T-Cell Senescence and SCI

In elderly individuals, the peripheral T-cell compartment undergoes marked remodeling: naïve T-cell numbers decline, while memory subsets—particularly CD45RA^+^ TEMRA cells—increase, impairing antigen recognition and clearance capabilities [75]. This compositional shift results in reduced antigenic response diversity and significantly diminished cytotoxic function of CD8^+^ T cells. Studies have shown that in aged individuals, memory CD8^+^ T cells exhibit reduced proliferative capacity and diminished cytotoxicity against viral and tumor targets [76, 77]. Additionally, IL-7 is a key cytokine for T-cell survival and proliferation; however, aging is associated with downregulation of IL-7 receptor expression, impairing the responsiveness of aged T cells to IL-7 signaling [78]. Tregs are expected to suppress excessive inflammation and promote tissue repair by secreting inhibitory cytokines such as IL-10 and TGF-β. However, aging compromises their functional efficacy. Studies have shown that aging leads to hypermethylation of the FoxP3 promoter and downregulation of CD25 expression in Treg cells, impairing IL-2/STAT5 signaling, suppressing their proliferation and immunosuppressive function [79]. Recent studies have revealed a marked decline in the intrinsic reparative capacity of aged Treg cells: they fail to promote remyelination or upregulate pro-repair genes effectively, and instead tend to co-express RORγt and IL-17, exhibiting an “inflammatory Treg” phenotype [33, 80]. These cells shift toward secreting cytokines such as IL-17 and IL-22, disrupting the microenvironmental stability at the injury site. For example, aged Tregs exhibit intrinsic functional impairment in lung injury models, resulting in a loss of reparative capacity [81]. However, exposure to a youthful microenvironment can partially restore their regenerative function [81]. Cutting-edge studies have demonstrated that low-dose IL-2 stimulation or CAR-Treg gene modification can remodel FoxP3 imprinting, restoring Treg stability and suppressive function in animal models [82, 83]. These approaches offer new avenues for immune cell therapy in elderly SCI. Similarly, in models of nervous system injury, such as multiple sclerosis (MS), aging has been shown to limit the regenerative capacity of Tregs, suggesting that Treg-mediated repair is also likely compromised in elderly patients with SCI [33].

### 5.1.2 B-Cell Senescence and SCI

Regarding B cells, aging similarly leads to a decline in humoral immunity. The number of B cells decreases in elderly individuals, and both the quantity and affinity of antibody secretion are diminished, resulting in weakened immune responses [84]. Studies have shown that aging promotes the accumulation of "age-associated B cells", which not only secrete pro-inflammatory cytokines such as TNF-α and IFN-γ but may also disrupt normal immune regulation [85, 86]. Further findings reveal that T-bet^+^CD11c^+^ age-associated B cells suppress somatic hypermutation and class-switch recombination by downregulating the expression of activation-induced cytidine deaminase, significantly reducing antibody affinity and diversity [87]. The aging-associated overactivation of the BAFF/BAFF-R signaling pathway and dysregulation of the PI3K/Akt pathway further enhance the pro-inflammatory phenotype of B cells and increase their resistance to apoptosis [88]. Animal model studies have shown that using BAFF-neutralizing antibodies can partially restore BCR diversity and humoral immune function, offering a potential strategy for antibody-mediated repair in elderly SCI patients [89]. In summary, immunosenescence places elderly patients with SCI at a significant disadvantage in terms of post-injury inflammation control and tissue repair. T cells exhibit reduced capacity to recognise and clear neural antigens, Tregs lose their anti-inflammatory and reparative functions, and B cells generate insufficient beneficial antibodies against injured tissue. These deficits contribute to the persistence of chronic inflammation and diminished regenerative outcomes following SCI. To counteract these mechanisms, several intervention strategies have been proposed. For example, IL-7 supplementation has been explored to enhance the survival and proliferation of aged T cells, while exogenous IL-7 stimulation may help expand both the naïve and memory T-cell pools [75].

On the other hand, broadening or transplanting functionally enhanced regulatory Tregs to restore their capacity to suppress inflammation and promote tissue repair represents another promising therapeutic approach. For instance, studies have shown that restoring a youthful gene expression profile in Tregs can enhance the reparative capacity of aged organisms [33]. In conclusion, interventions targeting age-related immune dysfunction—such as IL-7 therapy and Treg-based treatments—may offer promising avenues to improve inflammatory regulation and enhance recovery potential following SCI in older people.

### 5.2 Inflammaging and Barrier Dysfunction

Elevated systemic levels of inflammatory mediators in aged individuals can compromise the integrity of the blood-spinal cord barrier (BSCB), facilitating the breakdown of local immune regulation and promoting uncontrolled neuroinflammation. Elderly individuals and aged animal models often exhibit low-grade chronic systemic inflammation, known as "inflammaging," characterised by a sustained elevation of cytokines such as IL-6 and TNF-α. Cerebrovascular studies have shown that aging impairs mitochondrial function in brain endothelial cells and elevates IL-6 levels, directly compromising the integrity of the tight junctions within the blood-brain barrier [90]. In vitro experiments have demonstrated that exogenous IL-6 can mimic aging-related effects by suppressing mitochondrial function in brain endothelial cells and downregulating the expression of key tight junction proteins, such as claudin-5 [90]. It is reasonable to infer that the BSCB is similarly affected: age-associated elevation of IL-6, TNF-α, and other pro-inflammatory factors may increase BSCB permeability by promoting endothelial inflammation and disrupting tight junction integrity. In addition, SASP secretion by senescent glial cells—including IL-6, IL-1β, and matrix metalloproteinases such as MMP-3 and MMP-9—continuously erodes microenvironmental stability [74]. Studies have shown that astrocyte-derived IL-6 and MMPs can degrade tight junction proteins of the blood-brain barrier and upregulate cell adhesion molecules, thereby exacerbating barrier disruption [91, 92]. It is thus reasonable to infer that, under aging conditions during SCI, similar glial-derived SASP factors may also compromise the integrity of the BSCB. Animal models of SCI suggest that, compared to their young counterparts, aged animals exhibit a greater propensity for vascular barrier disruption and inflammatory cell infiltration [93, 94]. To address this issue, strategies aimed at suppressing inflammatory pathways and improving metabolic regulation may be employed. For instance, pharmacological agents that inhibit SASP-related factors, such as small molecules targeting the IL-6, IL-1, or NF-κB signalling pathways, or the use of mTOR inhibitors like rapamycin have shown potential to attenuate inflammation [95]. A study on inflammaging animal models found that treatment with rapamycin significantly reduced cerebrovascular permeability, restored the expression of tight junction proteins, and alleviated the overall inflammatory burden [95].

Additionally, enhancing systemic metabolic capacity, such as supplementation with NAD^+^ precursors, modulation of calcification regulators, or activation of AMPK signalling, has also been shown to reduce systemic inflammation [96-98]. Regarding lifestyle interventions, regular aerobic and resistance exercise have been shown to reduce circulating levels of pro-inflammatory cytokines, such as IL-6 and TNF-α, while also strengthening the structural integrity of the blood-brain barrier [99]. Therefore, integrated interventions, including SASP inhibitors, metabolic modulators, and regular physical exercise, promise to mitigate age-related systemic inflammation, enhance BSCB stability, and limit the spread of inflammation following SCI.

### 5.3 Impaired Inflammation Resolution

Aging impairs the ability of CNS-resident macrophages and microglia to clear apoptotic and necrotic cells, thereby prolonging the duration of inflammation [100]. Following SCI, the timely clearance of cellular debris resulting from widespread cell death is essential to terminate the inflammatory response and initiate tissue repair. Under aging conditions, both peripheral macrophages and central microglia exhibit significantly reduced phagocytic activity and impaired efferocytosis—the clearance of apoptotic cells. Recent studies have shown that in naturally aged mice, microglia display a diminished capacity to clear debris, such as β-amyloid, accompanied by downregulation of multiple phagocytic receptors, including TREM2 [101]. Compared to their younger counterparts, aged microglia exhibit widespread dysregulation of gene expression and are less effective at clearing neurotoxic protein deposits [95, 96]. Similarly, efferocytosis by aged macrophages is also impaired. One study reported that in an aged mouse model of hepatic ischemia-reperfusion injury, MerTK-mediated efferocytosis by macrophages was significantly impaired [102]. This defect is partly attributed to increased ADAM17 activity during aging, which promotes proteolytic cleavage of the MerTK receptor, impairing its function and establishing a negative feedback loop [102]. As a result, increased retention of apoptotic cells and accumulation of extracellular DNA are observed in the aged liver, leading to sustained activation of the STING pathway in macrophages [102]. In the context of SCI, similar mechanisms hinder the ability of aged macrophages and microglia to effectively clear apoptotic neurons and myelin debris, thereby perpetuating a pro-inflammatory environment. As a result, aged individuals often experience a prolonged inflammatory phase and more extensive secondary injury. Targeting this pathology by enhancing efferocytosis and debris clearance may offer a promising therapeutic strategy. For example, activating MerTK or inhibiting its proteolytic cleavage has been shown to significantly improve the efferocytic capacity of aged macrophages and mitigate inflammatory damage [103].

Additionally, using senolytic agents—such as Navitoclax—targeting aged immune cells has shown promising potential in restoring immune function and reducing inflammation [104]. In mouse models of SCI, clearance of senescent cells has led to significant improvements in motor and sensory function, enhanced preservation of spinal cord myelin, and reduced fibrotic scar formation and inflammatory cytokine secretion [72]. These findings suggest that senolytics can eliminate chronically activated, dysfunctional immune cells, promoting inflammation resolution and tissue repair. In summary, to address age-related impairments in phagocytic function, future strategies may involve a combination of immune cell function enhancement and senescent cell clearance, such as promoting MerTK signalling in macrophages, targeting the STING pathway, or administering senolytic agents, to accelerate inflammation resolution and recovery following SCI.

## 6. Challenges and Therapeutic Prospects

This section discusses the unique challenges faced by elderly spinal SCI patients in anti-inflammatory treatment, particularly highlighting how chronic inflammation caused by aging severely affects the patients' recovery outcomes (Table 2). Table 2 summarizes anti-inflammatory therapy's significant challenges and prospects in elderly patients with spinal cord injury (SCI), focusing on current research gaps and difficulties in applying anti-inflammatory strategies to aging models. It details innovative therapeutic approaches targeting the inflammatory response, including IL-1RA, NLRP3 inhibitors, senolytic agents, exosome- and miRNA-based therapies, exploring their mechanisms of action and potential, while highlighting the lack of validation in aged SCI models. The table also outlines key research issues, such as the absence of standardized aging animal models and insufficient evidence on the efficacy and safety of anti-inflammatory drugs in elderly populations. Finally, it advocates for accelerating the development of personalized therapeutic strategies, incorporating biomarkers and multi-omics dynamic monitoring, and integrating pharmacological, rehabilitative, and nutritional interventions to achieve precision medicine and improve treatment outcomes and quality of life in elderly SCI patients. While recent years have seen the emergence of some innovative anti-inflammatory strategies, such as IL-1 receptor antagonists (IL-1RA), NLRP3 inflammasome inhibitors, and senolytic drugs, which have shown particular efficacy in young animal models, the effectiveness and safety of these therapies in aged SCI animal models remain insufficiently validated. Furthermore, the commonly used anti-inflammatory drugs in clinical settings generally exhibit limited efficacy and potential safety risks in the elderly patient population. Therefore, developing anti-inflammatory drugs specifically targeting elderly patients urgently needs to be advanced to address the gap between current research and clinical practice effectively.

### 6.1 Anti-Inflammatory Therapy for SCI in the Elderly: Current Challenges and Research Gaps

We contend that current research on SCI significantly underestimates the unique inflammatory challenges associated with aging. With a marked increase in the proportion of elderly patients with SCI, age-related chronic inflammation has been shown to exacerbate secondary injury processes, leading to significantly poorer functional recovery compared to younger individuals [105].

**Table 2:** Therapeutic Challenges and Promising Interventions for Neuroinflammation in Aged SCI.

Section	Challenges and Research Gaps	Promising Therapeutic Strategies	Mechanisms of Action	Current Research and Future Directions	References
**Challenges in Anti-Inflammatory Therapy** **(6.1)**	-Aging-related chronic inflammation has been underestimated -Traditional anti-inflammatory drugs have limited efficacy and high safety risks in aged SCI -Lack of standardized aged animal models			-Establishing standards for aged SCI models -Enhancing drug efficacy/safety evaluation in aged models	[29, 105-109]
**Innovative Anti-Inflammatory Intervention Strategies** **(6.2)**	-IL-1RA, NLRP3 inhibitors, and senolytic drugs lack validation in aged SCI models	- IL-1RA - MCC950 (NLRP3 inhibitor) - Senolytics	- Inhibition of IL-1 signaling - Blocking NLRP3 inflammasome - Selective clearance of senescent cells	- Systematically validate efficacy/safety in aged SCI animals - Optimize dosage and administration regimen	[72, 110-116]
**Regulation of Microglial Phenotypes** **(6.3)**	- Long-term systemic intervention safety is unknown - Significant differences across strains/dosages	- PPARγ agonists (e.g., pioglitazone) - mTOR inhibitors (e.g., rapamycin)	- Promote microglial polarization towards the M2 phenotype - Restore autophagy and clear damaged cell debris	- Evaluate cardiovascular and immune side effects in aged models - Optimize targeted delivery to reduce systemic exposure risks	[117, 118]
**Exosome-based and miRNA Therapies** **(6.4)**	- Lack of long-term efficacy and safety data in aged models	- Neuron/MSC-derived exosomes carrying miR-124-3p, miR-125, etc.	- Inhibit the PI3K/AKT/NF-κB pathway, reduce M1/A1 glial cell activity, and alleviate inflammation	- Conduct in-depth research on aged animal models - Optimize exosome preparation and administration routes, and evaluate immunogenicity	[119-121]
**Rehabilitation and Nutritional Interventions** **(6.5)**	- Lifestyle intervention studies in aged SCI are not standardized - Lack of standardized data	- Regulated rehabilitation training -ω-3 fatty acids/antioxidant diet - Restrictive diet/intermittent fasting	- Reduce systemic/central inflammation through the muscle-brain axis - Enhance mitochondrial function and antioxidant defense	- Conduct rigorous clinical studies to validate the safety and efficacy of interventions -Develop rehabilitation/nutritional intervention guidelines for aged SCI	[122-128]
**Challenges and Future Perspectives in Precision Medicine Approaches** **(6.6)**	- Insufficient biomarkers and stratification standards - Limited data on elderly clinical patients, with high heterogeneity - Animal models are predominantly young	- Biomarker-guided personalized combination interventions	- Multi-omics dynamic monitoring for precise matching of drug, rehabilitation, and nutrition plans	- Establish a multicenter aged SCI database and RWE - Develop "Consensus Guidelines for Aged SCI Models" - Develop a dynamic decision support system	[129-137]

Animal model studies consistently demonstrate that aged SCI models exhibit more pronounced and sustained activation of microglia and astrocytes, intensifying glial scar formation and severely impairing the potential for neural regeneration [106]. However, most current anti-inflammatory research is still focused on young animal models, and this bias limits our understanding of the pathological mechanisms underlying aging SCI [29]. Due to the unique impact of aging on the immune system and neural regeneration processes, existing studies often fail to adequately reflect the actual condition of elderly patients [107]. Additionally, the standardisation of aged SCI animal models remains a significant issue that needs to be addressed. For example, a lack of unified standards for constructing aged models across different laboratories leads to incomparable results between studies, affecting the cross-age applicability of anti-inflammatory treatment strategies [108]. Therefore, future research should focus on standardising aged SCI models to provide more reliable data support for clinical applications. Additionally, commonly used anti-inflammatory drugs in clinical practice, such as high-dose methylprednisolone, have limited efficacy in elderly patients and are associated with significant safety issues [109]. Therefore, there is a severe lack of anti-inflammatory drug development specifically targeting elderly SCI individuals, and there is a noticeable gap in research focusing on aging SCI models and individual differences. These issues limit the effective translation and application of anti-inflammatory treatment strategies in elderly patients.

### 6.2 Innovative Anti-Inflammatory Interventions and Their Underlying Mechanisms

Although effective in younger models, we highlight several promising anti-inflammatory approaches that have yet to be adequately investigated in the context of SCI in older people. Various novel anti-inflammatory interventions have been explored in SCI models in recent years. For example, IL-1RA has effectively suppressed acute inflammatory responses in young SCI animal models by reducing the infiltration of neutrophils and other inflammatory cells into the spinal cord lesion [110]. However, the systemic immunosuppressive effects of IL-1RA may increase the risk of infection and potentially delay wound healing in elderly individuals [111, 112]. Additionally, its pharmacokinetics in elderly populations remains insufficiently characterized. Moreover, existing research is primarily conducted in adult mice, lacking a deep understanding of immune system changes during aging. The assessment of elderly SCI individuals needs to consider differences in immune responses and address changes brought about by aging, such as metabolic, endocrine, and tissue remodelling alterations [113]. Furthermore, most studies on anti-inflammatory interventions remain at the stage of using young models and fail to fully consider the specific needs of aged models. Therefore, future research should strengthen the assessment of elderly SCI individuals and develop anti-inflammatory intervention strategies tailored to aging characteristics. Similarly, NLRP3 inflammasome inhibitors, such as MCC950, have significantly reduced pro-inflammatory cytokine levels and improved functional recovery after SCI in young mice by blocking the formation of the NLRP3 complex [114]. This approach has been validated in acute SCI animal models. However, in aged models, chronic administration of NLRP3 inflammasome inhibitors may induce hepatotoxicity, nephrotoxicity, or yet undefined off-target effects, and their safety window and optimal dosing require validation through comprehensive toxicological and pharmacokinetic studies [115]. Senolytic drug strategies targeting senescent cells have also garnered increasing attention. Recent studies have demonstrated that the selective elimination of senescent cells within the scar tissue of SCI lesions, using specific senolytic agents, can suppress the secretion of pro-fibrotic and pro-inflammatory factors, significantly attenuate inflammation, and subsequently enhance the recovery of motor, sensory, and bladder functions [72]. However, senolytics, while clearing excessive senescent cells, may inadvertently damage healthy cells and provoke tissue dysfunction [116]. Its safety and tolerability in elderly subjects with multiple comorbidities remain unsupported by sufficient clinical or animal data. These findings suggest that senescent cells are critical in sustaining chronic inflammation following SCI. However, further research is needed to selectively eliminate these cells without compromising normal immune function. We strongly advocate for evaluating these interventions in aged animal models as a priority before advancing to clinical translation.

### 6.3 Regulation of Microglial Phenotypes

We propose that targeted modulation of microglial phenotypes represents a promising strategy for mitigating the exacerbated inflammatory response following SCI in the aging population. PPARγ agonists, such as pioglitazone, have been shown in young animal models to promote microglial polarisation toward the anti-inflammatory M2 phenotype, thereby protecting neurons and enhancing motor function [117]. However, systemic activation of PPARγ may also induce cardiovascular and other adverse effects, and its efficacy appears to be sensitive to factors such as animal strain and dosage. Therefore, further validation is needed in the context of aging. Restoration of autophagic function is thought to alleviate inflammation by clearing pathological protein aggregates from damaged cells. Studies using the mTOR inhibitor rapamycin to treat SCI in mice have shown that it significantly enhances Beclin1 expression and autophagosome formation at the injury site [118]. Rapamycin treatment reduced neuronal apoptosis and improved functional recovery in treated animals by promoting autophagic clearance of damaged mitochondria and cellular debris. However, excessive activation of autophagy may pose potential risks, and mTOR inhibition exerts complex effects on the immune system. These limitations warrant careful evaluation in future studies.

### 6.4 Exosome-Based and microRNA (miRNA) Therapies

Emerging evidence suggests that exosome-mediated delivery of miRNAs represents a potent strategy for modulating neuroinflammation. Exosomes derived from neurons or stem cells can transport miRNAs and other bioactive molecules across the blood-brain barrier, allowing for targeted regulation of inflammatory pathways within the spinal cord. Neuron-derived exosomes carrying miR-124-3p have effectively suppressed the pro-inflammatory M1 microglia and neurotoxic A1 astrocyte activities, thereby reducing inflammation and promoting functional recovery [119]. This process is partially mediated through inhibition of the PI3K/AKT/NF-κB signalling pathway, ultimately contributing to functional recovery. Similarly, exosomes derived from mesenchymal stem cells, enriched in miRNAs such as miR-124 and miR-125, have also demonstrated notable anti-inflammatory potential [120, 121]. However, data on the efficacy and long-term safety in aged SCI animal models remain lacking. Further in-depth studies are urgently needed to assess their potential for clinical translation.

### 6.5 The Impact of Rehabilitation and Nutritional Interventions on Neuroinflammation

We emphasise that rehabilitation and nutritional interventions are not merely supportive measures but critical modulatory strategies capable of directly regulating chronic inflammatory pathways exacerbated by aging. Evidence indicates that regular physical exercise releases skeletal muscle-derived signalling molecules, known as myokines, which influence the CNS via the muscle-brain axis. Exercise-induced myokines have been shown to promote neurogenesis, angiogenesis, and synaptic plasticity within the brain [122]. Accordingly, rehabilitation training in patients with SCI has been associated with reductions in common inflammatory markers, such as C-reactive protein and IL-6, enhanced mitochondrial function and antioxidant defences, thereby ameliorating delayed neural injury [123, 124]. Meanwhile, dietary factors and metabolic modulators have also attracted considerable attention. For example, anti-inflammatory diets rich in omega-3 fatty acids, antioxidant vitamins, and polyphenols are believed to help attenuate inflammation and support neural repair [125-127]. Early animal studies have also shown that caloric restriction or intermittent fasting can enhance neuronal survival and modulate glial cell responses [128]. Although these lifestyle intervention strategies are generally considered safe, standardised investigations in aged SCI populations remain limited and require more rigorous clinical studies to validate their efficacy.

### 6.6 Challenges and Future Perspectives in Precision Medicine Approaches

As the proportion of elderly SCI patients continues to rise, the highly heterogeneous pathological characteristics and recovery patterns of this population pose greater demands on precision medicine. In current research, insufficient biomarker development, unclear patient stratification standards, and the over-reliance on young animal models severely limit the progress of personalised treatment strategies for elderly SCI individuals, requiring systematic improvements.

### 6.6.1 Biomarker Development and Dynamic Monitoring

The pathological response in elderly SCI patients is complex and requires a combination of multi-type, multi-level biomarkers. In addition to common biomarkers, it is essential to integrate proteins from blood and cerebrospinal fluid, metabolites, cytokines, and miRNAs. This multifaceted approach will provide a more comprehensive understanding of disease progression and response to treatment, enabling more accurate patient stratification and personalised therapeutic interventions. A combined screening approach involving genomics, transcriptomics, proteomics, and metabolomics is recommended. It is also recommended to dynamically monitor the changes in their concentrations at different time points (acute, sub-acute, and chronic) to assess the injury progression and treatment response [129]. This comprehensive strategy will enable a more accurate evaluation of the biological processes underlying SCI and the effectiveness of therapeutic interventions, facilitating the development of personalised treatment plans for elderly SCI patients. Standardised detection procedures—including sample processing, assay kits, and analytical software—and multicenter validation can enhance result consistency and provide precise evidence to support clinical decision-making [130].

### 6.6.2 Patient Stratification Strategy and Clinical Trial Design

Given the high heterogeneity of elderly patients, clinical trials must implement refined stratification [131]. Biological age (e.g., DNA methylation clock) should be integrated with chronological age, categorising individuals into age groups of 60-69, 70-79, and ≥80 years. Stratification should be based on the ASIA impairment scale (complete/incomplete injury). Comorbidity burden (including cardiovascular diseases, diabetes, and osteoporosis) and frailty scores should also be considered. The study design should employ stratified randomisation and covariate adjustment, with dual stratification based on 'clinical features + molecular profile' and biomarker levels at baseline [132, 133]. Multicenter, adaptive design can balance sample sizes across subgroups and optimise recruitment and intervention strategies based on interim analysis [134].

### 6.6.3 Limitations of the Young Model and Standardization of the Aging Model

Previous young animal models have overestimated treatment effects, neglecting age-related chronic inflammation and tissue degenerative changes. The aging model should use rats aged ≥18 months (equivalent to middle-aged humans), with blunt impact or compression injury at the cervical or thoracic level to simulate clinical incomplete SCI. In addition to the BBB score, functional assessments should include the CatWalk gait analysis, sensory threshold testing, and consideration of the impact of baseline motor function decline [135]. Surgical care should be adapted for older animals to reduce postoperative mortality [136]. To enable comparability of data across different laboratories, establishing a "Consensus Guidelines for Aging SCI Models" is recommended, specifying animal age, injury parameters, assessment indicators, and sex ratio.

### 6.6.4 Clinical Data Scarcity and Acquisition of Multicenter Real-World Evidence

Existing studies often exclude elderly patients, resulting in insufficient data for the elderly population and survivor bias [137]. To fill the gap, a multicenter elderly SCI database should be established to prospectively collect demographic data, injury characteristics, treatment protocols, and long-term outcomes, while standardising the definitions of complications and follow-up indicators. By integrating electronic medical records, big data, and wearable device monitoring, real-world evidence (RWE) can be obtained to validate clinical trial results' external validity and safety. Furthermore, these clinical data should be cross-referenced with biomarkers and intervention targets identified in aging animal models to facilitate their translation.

### 6.6.5 Future Perspectives on Precision Management of Personalised Combined Interventions

Treatment of elderly SCI requires a comprehensive approach combining "medication + rehabilitation + nutrition." Regarding medication, IL-1RA, MCC950, or Senolytics should be selected based on the stratified patients' inflammatory load and biological characteristics, combined with mTOR modulation or PPARγ agonists [110, 114, 117, 118]. In terms of rehabilitation, a functional training regimen combining low-intensity, high-frequency, and intermittent high-intensity exercises should be developed [122]. In terms of nutrition, assess and correct malnutrition by providing individualised supplementation with high-protein, ω-3 fatty acids, and antioxidants [125-127]. A decision support system can be established through multi-omics data and dynamic monitoring to achieve "treatment, monitoring, and adjustment in parallel," thereby continuously optimising the intervention combination [129]. For different elderly subtypes, the optimal plan can be precisely matched to maximise functional recovery and improve quality of life.

## 7. Conclusion and Prospects

In conclusion, chronic neuroinflammation plays a central role in the long-term pathophysiology of SCI, and its impact is particularly profound in elderly individuals. The aging process exacerbates inflammation by altering immune responses, reducing the capacity for inflammation resolution, and compromising cellular functions, all hindering neural repair and functional recovery. As the global population ages, the incidence of SCI in elderly individuals is expected to increase, and understanding the unique inflammatory mechanisms in this demographic is essential for developing effective treatment strategies.

Current research primarily relies on young animal models, which fail to fully represent the complexities of aging, and the distinct inflammatory profiles observed in elderly SCI patients. This has led to a significant gap in translating therapeutic interventions from preclinical studies to clinical practice. Therefore, developing standardised aged SCI models is critical to addressing the specific challenges of older patients and advancing our understanding of how aging affects post-injury inflammation and repair.

Recent advancements in immunology, molecular biology, and regenerative medicine provide promising avenues for developing targeted therapies to mitigate chronic neuroinflammation in elderly SCI patients. Approaches such as senolytic therapies, NLRP3 inflammasome inhibitors, and exosome-based treatments hold potential for addressing the inflammatory aspect of SCI and promoting tissue repair and functional recovery. Modulating microglial phenotypes, enhancing autophagy, and promoting inflammation resolution through strategies such as activating MerTK signalling or using metabolic modulators may offer new ways to mitigate the persistent inflammation that limits recovery in older individuals.

The integration of precision medicine into SCI treatment, particularly for the aging population, will require a multi-faceted approach that includes pharmacological interventions, gene therapy, rehabilitation, and lifestyle modifications. It will also necessitate the development of reliable biomarkers for better patient stratification, enabling personalised treatment plans that optimise therapeutic outcomes. Advancements in multi-omics technologies, dynamic monitoring of inflammation, and developing more accurate animal models will be essential in guiding these personalised approaches.

Looking ahead, more rigorous clinical trials focused on elderly SCI patients are needed to validate the efficacy and safety of these novel therapeutic interventions. Additionally, integrating insights from aging research into the development of anti-inflammatory drugs and rehabilitation protocols specifically designed for older people will be critical for improving the prognosis of SCI in this population. A shift toward age-tailored treatments, supported by robust preclinical and clinical data, will significantly enhance recovery outcomes and quality of life for elderly SCI patients.

In summary, addressing the unique challenges posed by chronic neuroinflammation in aging SCI patients is an urgent and complex task. However, with the growing understanding of aging-related inflammatory mechanisms and the emergence of innovative therapeutic approaches, there is hope for more effective, age-specific treatments that can improve the functional recovery and overall well-being of elderly individuals suffering from SCI.
